# Reply to Piochon et al.: NMDARs in Purkinje cells are not involved in parallel fiber–Purkinje cell synaptic plasticity or motor learning

**DOI:** 10.1073/pnas.2120480119

**Published:** 2022-02-22

**Authors:** Martijn Schonewille, Allison E. Girasole, Chris I. De Zeeuw, Guy Bouvier

**Affiliations:** ^a^Department of Neuroscience, Erasmus Medical Center, NL-3000 CA Rotterdam, The Netherlands;; ^b^Neuroscience Graduate Program, University of California, San Francisco, CA 94158;; ^c^Department of Neurology, University of California, San Francisco, CA 94158;; ^d^Kavli Institute for Fundamental Neuroscience, University of California, San Francisco, CA 94158;; ^e^Weill Institute for Neurosciences, University of California, San Francisco, CA 94158;; ^f^Netherlands Institute for Neuroscience, Royal Dutch Academy of Arts and Sciences, NL-1000 GC Amsterdam, The Netherlands;; ^g^Institut de Biologie de l’Ecole Normale Supérieure, Ecole Normale Supérieure, Université PSL, 75005 Paris, France;; ^h^CNRS, UMR 8197, 75005 Paris, France;; ^i^INSERM, U 1024, 75005 Paris, France

In our study (1), we presented functional and immunohistochemical evidence for the presence of presynaptic *N*-methyl-D-aspartate receptors (pre-NMDARs) at parallel fiber–Purkinje cell (PF-PC) synapses in the adult cerebellum. As in young rodents, pre-NMDARs are required to induce PF-PC synaptic plasticity (2). Using cell-specific deletion of NMDARs in granule cells (GCs) or PCs, we demonstrated that only GC NMDARs are robustly involved in PF-PC synaptic plasticity and vestibuloocular reflex (VOR) adaptation.

Our data contradict those by Piochon et al. (3), who proposed that PC NMDARs initiate PF-PC long-term depression (LTD). The question remains to what extent PC NMDARs at climbing fiber (CF) synapses may affect PF synapses. The amplitude of CF-PC NMDA currents is orders of magnitude smaller than the AMPA currents that cause a depolarized plateau. It is thus unlikely that CF-PC NMDARs can directly affect electrogenesis during complex spikes. However, blockage of CF-PC NMDARs could still affect PF-PC LTD through long-term effects on PC excitability and CF dendritic spikes (4) or CF-PC LTD (5).

Piochon et al. (3) partly based their conclusions on blocking PC NMDARs with intracellular MK801 and performing direct PF stimulation in sagittal slices at high calcium concentrations. Since the MK801 concentration used in the intracellular medium is 1,000 times higher than the concentration required in an extracellular medium, MK801 may have leaked out of the cell (or the pipette prior to patching), blocking pre-NMDARs on PFs. Moreover, their stimulation configuration may have bypassed involvement of pre-NMDARs in LTP induction and changed the plasticity rule by perturbing presynaptic calcium dynamics (1, 2). We, instead, used cell type–specific genetic deletions of NMDARs and transverse slices that allowed PF stimulation far away from the recording site, precluding the caveats described above.

Consistent with the concept that we proposed (6), Piochon et al. (7) suggest that, when using physiological concentrations of divalent cations, similar to the one in our study (1), LTD requires clusters of complex spikes. However, not only the stimulus pattern of CF activity (6) but also the presynaptic stimulation conditions should optimally match the in vivo situation. They used direct PF stimulation in sagittal slices that activates a bundle of axons, which is unlikely to occur in vivo and could affect PF-PC LTD induction (8).

With respect to the behavioral experiments, our previous work on mutant mouse lines, in which LTD is ablated, has indeed indicated that PF-PC LTD is not essential for VOR adaptation (9). Instead, PF-PC LTP and concomitant simple spike increases in firing rate appear to provide an essential contribution to VOR adaptation (9–11). Given that not only PF-PC LTD but also LTP is affected in the pre-NMDAR GC-GluN1 mice (1), we selected a behavioral paradigm that is linked to a lack of LTP. We, indeed, also found a phenotype in mice with PC-specific deletion of NMDARs, albeit subtle. As LTD was not impaired in these mice, this does not contradict our interpretation. It could reflect a role for PC NMDARs, such as in plasticity of CFs, which we did not study here.

Taken together, we appreciate the feedback of Piochon et al. (7) and share their drive to gain more insight through further studies. However, based on our findings and the current literature, we do not see sufficient reason to change our original conclusions.
